# Protracted, Intermittent Outbreak of *Salmonella* Mbandaka Linked to a Restaurant — Michigan, 2008–2019

**DOI:** 10.15585/mmwr.mm7033a1

**Published:** 2021-08-20

**Authors:** William D. Nettleton, Bethany Reimink, Katherine D. Arends, Douglas Potter, Justin J. Henderson, Stephen Dietrich, Mary Franks

**Affiliations:** ^1^Kalamazoo County Health and Community Services Department, Kalamazoo, Michigan; ^2^Department of Family and Community Medicine, Western Michigan University Homer Stryker M.D. School of Medicine, Kalamazoo, Michigan; ^3^Michigan Department of Health and Human Services.

In 2018, Michigan public health officials determined that a single restaurant in southwest Michigan was the source for a protracted, intermittent outbreak of *Salmonella* enterica serotype Mbandaka infections occurring since 2008. Isolates from 36 infected persons shared two highly related pulsed-field gel electrophoresis (PFGE) patterns and highly related whole genome sequencing (WGS) subtypes. The initial focus of the local public health investigation on food items rather than food sources (i.e., restaurants) through a questionnaire, difficulty in food history recollection among ill persons, and sporadic case identification over periods from months to years contributed to delayed source identification. The Kalamazoo County Health and Community Services Department (KHCSD) and the Michigan Department of Health and Human Services (MDHHS) collected clinical specimens, performed multiple rounds of environmental testing, and conducted multiple regulatory visits, and based on accumulated findings over 10 years, identified the restaurant source. A 2018 investigation by KCHCSD and MDHHS found that environmental samples and stool specimens from asymptomatic restaurant employees tested positive for the *Salmonella* Mbandaka outbreak strain. A complex association between the restaurant environment and employees resulted in patron illnesses. Environmental health interventions, facility renovation, asymptomatic employee exclusions, employee health monitoring, and recurrent facility environmental sampling measures were implemented. As a result of ongoing cases and environmental persistence of *Salmonella* Mbandaka, the restaurant closed permanently in 2018. Restaurant employee stool testing and environmental sampling for *Salmonella* early during the investigation of confirmed *Salmonella* cases linked to a restaurant enhances source identification. Exclusion or restriction of asymptomatic food workers with stool-positive nontyphoidal *Salmonella* should be considered part of restaurant outbreak mitigation.

## Epidemiologic Investigation

In 2012, KCHCSD was notified by MDHHS about *Salmonella* Mbandaka cases occurring intermittently since 2008 that were highly related by PFGE pattern. During 2012–2014, a restaurant was not yet associated, so a hypothesis-generating questionnaire was used to ensure capture of detailed patient food histories, which included closed-ended questions about frequently eaten food items, types of restaurants visited, and animal contact. In 2014, although investigations into common suppliers among several restaurants mentioned in food histories were ongoing and other restaurants were named by cases, as more information was collected from supplementary questionnaires, KCHCSD, MDHHS, and the Michigan Department of Agriculture and Rural Development discussed the association of a single restaurant (restaurant A) based on five known, confirmed cases to date reporting a meal at restaurant A. KCHCSD gathered additional information from restaurant A management regarding employee health and exposures, facility and equipment, food sources, and pest control. No further epidemiologic link was established from the interview with the restaurant, but continued cleanliness and maintenance citations occurred during 2014–2018. Additional *Salmonella* Mbandaka cases in 2017 prompted development of a detailed, outbreak-specific case questionnaire that included specific questions about restaurant A. As more cases were identified, an intensive investigation began in 2018. An outbreak case was defined as a case of confirmed *Salmonella* Mbandaka with one of two closely related PFGE patterns (TDRX01.0120 and TDRX01.0127), highly related WGS subtype identified by the MDHHS Bureau of Laboratories or CDC, or probable cases with clinically compatible illness and epidemiologic linkage to a confirmed case.

During September 2008–July 2019, a total of 35 primary cases (33 confirmed and two probable) and one confirmed secondary case were identified. Patients with confirmed cases ranged in age from 1.5–90 years (mean = 57 years; median = 64 years), and 26 (72%) patients were female. Several patients reported a history of chronic gastrointestinal issues that made determination of onset date difficult. Twenty-four (67%) patients reported vomiting or diarrhea, and 12 (33%) reported urinary tract infection. Six (17%) patients were hospitalized. Approximately 40% of patients had underlying medical conditions such as diabetes or cancer. Among 19 patients with a restaurant dining history, 17 reported eating at restaurant A. Patients were routinely interviewed at the time of local health department referral and reinterviewed, often weeks later, when Mbandaka serotype was reported. Thirteen patients, retrospectively identified from early in the outbreak period, were not candidates for reinterview because their onsets preceded identifying them as part of the outbreak by >1 month.

After implementation of the outbreak-specific questionnaire in 2017, nine patients with onset during August 2017–July 2019 reported having eaten at restaurant A (Figure). To determine whether restaurant A was mentioned in the food histories of other reported foodborne illnesses, Michigan public health officials reviewed restaurant A patronage and food histories of 1,166 persons with previously reported salmonellosis, campylobacteriosis, and shigellosis cases in southwest Michigan for restaurant dining history. The only patients who reported eating at restaurant A were those associated with this outbreak; no other patients mentioned the restaurant.

**FIGURE:**
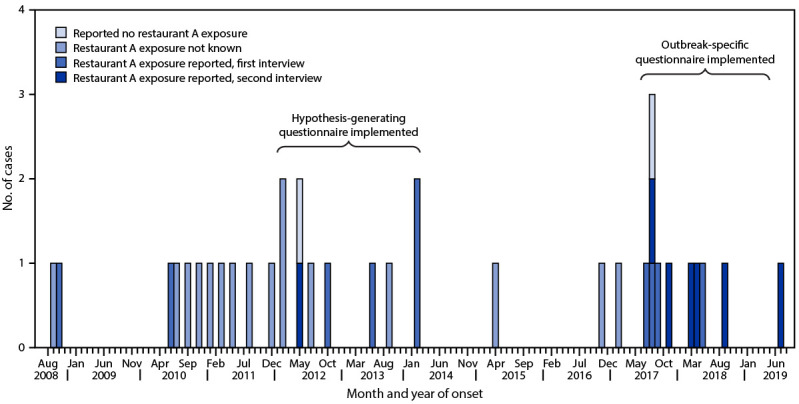
Cases of *Salmonella* Mbandaka outbreak subtype (N = 35), by month and year of illness onset* and restaurant A exposure — Michigan, September 2008–July 2019^†^ * Onset date was missing for five patients; for these cases, the date of referral to the health department was used. ^†^ Pulsed-field gel electrophoresis was performed only on the top 20 *Salmonella* serotypes submitted to the Michigan Department of Health and Human Services Bureau of Laboratories from approximately 2009 to early 2010; the *Salmonella* Mbandaka serotype was rare and not a top 20 serotype.

## Environmental and Laboratory Investigation

As part of the 2018 investigation, restaurant A employee stool specimens and environmental samples were collected in parallel and analyzed for *Salmonella*. None of the 100 employees reported symptoms at the time of sample collection or in the weeks preceding collection. MDHHS Bureau of Laboratories identified five isolates from four of 100 asymptomatic employees’ stool specimens that shared the outbreak subtype. Stool cultures were collected approximately every 30 days from asymptomatic employees with stool specimens that tested positive until a negative test result was received. A repeat stool culture was taken at least 48 hours after the first negative result. Asymptomatic employees who received positive *Salmonella* Mbandaka test results were required to have two negative stool cultures before returning to work. Because antibiotics can increase the likelihood of prolonged *Salmonella* shedding in stool, treatment was not recommended (*1*). None of the restaurant employees received antibiotics to treat asymptomatic *Salmonella* Mbandaka infection. The duration of *Salmonella* shedding among the four asymptomatic restaurant employees with positive cultures varied (range = 31–123 days).

A team of local and state public health officials collected 80 environmental samples from food and nonfood contact surfaces to be tested for *Salmonella*. *Salmonella* was isolated from 39 (49%) environmental samples. Positive samples shared the same PFGE and WGS results as the outbreak subtype (Table). Positive samples were collected throughout the restaurant kitchen, including cooking, preparation, dishwashing, storage, and employee restroom areas. Following the identification of a new case in September 2018, a second round of environmental *Salmonella* sampling was conducted. In the second round, *Salmonella* was isolated from 11 of 81 samples (14%) and shared the outbreak subtype. Positive environmental sites were generally similar, but not identical, in the two rounds. Environmental, asymptomatic employee, and symptomatic patient isolates identified by core genome multilocus sequence typing (cgMLST) revealed three clades (A, B, and C) (Table). Isolates within each clade were highly related, differing by ≤5 alleles. The clades were also considered highly related to each other, differing by ≤15 alleles. All environmental and employee isolates were in clade A; symptomatic patient isolates were identified in all three clades.

**TABLE:** Characteristics of *Salmonella* Mbandaka outbreak subtype isolates from symptomatic patients, asymptomatic restaurant A employees, and restaurant A environmental surfaces — Michigan, August 2008–June 2018

Source of isolate	No. of samples collected	No. (%) of isolates identified by PFGE	No. (%) of isolates identified by WGS	Isolation date or date range	Clade by cgMLST
Symptomatic patient	36	36 (100)	30 (83)	2008–2012	B
				2012–2014	C
				2015–2018	A
Asymptomatic employee*	100	5 (5)	5 (5)	Jun 2018	A
Environment (restaurant)	80	39 (49)	26 (33)	Jun 2018	A
Environment (restaurant)	81	11 (14)	10 (12)	Nov 2018	A

**Abbreviations:** cgMLST = core genome multilocus sequence typing; PFGE = pulsed-field gel electrophoresis; WGS = whole genome sequencing.

* Five isolates were analyzed from four asymptomatic employees.

## Public Health Response

In addition to routine inspections and administrative hearings in 2012 and 2014, iterative facility environmental assessments and administrative hearings during 2017–2018 addressed cleanliness, lack of active managerial control, and other foodborne illness risk factors cited at restaurant A. In addition to employee stool screening for *Salmonella* and exclusion of asymptomatic employees with *Salmonella* stool positive results, employees were also required to submit stool specimens for *Salmonella* testing if new onset of gastrointestinal symptoms occurred during the 2018 public health response. Seven employees reported symptoms, but all had negative *Salmonella* test results. Before the initial environmental *Salmonella* sampling event in spring 2018, the facility temporarily closed for renovations of the kitchen, flooring, walls, and major equipment. The restaurant was required to clean all facility and food contact surfaces to norovirus cleaning standards* and underwent follow-up environmental *Salmonella* sampling. In fall 2018, the facility temporarily closed for additional floor and equipment renovations with the intent of eradicating *Salmonella* from the facility. The facility again had a full norovirus standard cleaning performed before reopening. Despite kitchen renovation and environmental hygiene interventions, *Salmonella* Mbandaka continued to be detected in restaurant A. Therefore, the restaurant voluntarily and permanently closed in late 2018, and food, dishes, storage, soft goods, chairs, and tables were destroyed. The metal food production equipment was extensively cleaned, quarantined, and resampled for *Salmonella* before being redeployed. The building was deemed ineligible for food production or storage relicensure. One case of the outbreak subtype was isolated in urine 8 months after closure of the restaurant; the patient reported chronic, intermittent diarrhea after eating at restaurant A 3 weeks before it closed.

## Discussion

Multiple challenges contributed to delayed source identification. Food histories were incomplete in the early cases. Initial questionnaires were inflexible and focused more on food items than on food establishments. Early cases were not initially identified as a cluster given the sporadic incidence and were hypothesized as a rare or regional PFGE pattern. Further, index of suspicion for a protracted common source early in the outbreak was low given the more typical experience of point source *Salmonella* outbreaks. Finally, restaurant management was doubtful and required intensive engagement. Both the environmental and clinical testing results were thus essential for continued mitigation efforts.

Salmonellosis outbreaks in the food industry often occur through a point source when undercooked or contaminated food products infect consumers until distribution of the foodborne vehicle ceases (*2*,*3*). In this outbreak, a complex association between the environment and employees of a single restaurant in southwest Michigan demonstrated a protracted and intermittent common source outbreak of *Salmonella* Mbandaka. A study of 23 restaurant-associated salmonellosis outbreaks found that restaurants with *Salmonella*-positive environmental samples had a higher proportion of *Salmonella*-positive food workers and longer outbreak durations than did restaurants with negative environmental samples (*4*). The nearly 11-year duration of this outbreak attests to the potential recalcitrance of *Salmonella* in restaurant environments, the importance of hygienic restaurant policies and practices, and the challenge in source identification when cases occur intermittently and without a clear foodborne vehicle. As WGS is more broadly implemented as a routine subtyping method for *Salmonella* and other bacterial enteric pathogens, increased discriminatory power might facilitate the identification of more protracted, common-source outbreaks (*5*). Whereas initially small numbers of cases might present a challenge to definitively implicating a common source, gathering as much high quality exposure data as possible, including repeated interviewing of patients with cases that are clustered in time using closed-ended questions about exposures of interest, can aid an investigation. In addition, conducting environmental assessments, environmental sampling, and employee testing for *Salmonella* are best practices that should be considered early in an investigation, particularly when a single foodborne vehicle is not apparent.

Fifteen (42%) of the 36 patients had the outbreak subtype isolated in urine; 12 (33%) patients had urinary symptoms without reporting diarrhea or vomiting. These findings are consistent with the observation that a higher proportion of *Salmonella* serogroup C1 (including Mbandaka) than of other *Salmonella* serogroups is isolated from urine (*6*,*7*). Although chronic carriage of *Salmonella* Typhi after acute infection is widely recognized, asymptomatic carriage of nontyphoidal *Salmonella* is less well characterized but has been reported in restaurant food and hotel workers as well as in healthy adults and children (*5*,*8*,*9*).

For most of the time when the reported outbreak investigation was conducted, the restaurant was regulated under a modified version of the 2009 Food and Drug Administration (FDA) Food Code, the latest FDA Food Code that Michigan had adopted. The 2009 FDA Food Code did not include asymptomatic nontyphoidal *Salmonella* infections among the five specific foodborne pathogens^†^ for which exclusion and restriction requirements are delineated. Therefore, the 2017 FDA Food Code was used for guidance because it includes asymptomatic nontyphoidal *Salmonella* infection as a food worker condition of restriction (*10*). Further adoption of the 2017 FDA Food Code will aid public health professionals in disrupting nontyphoidal *Salmonella* transmission in restaurant settings, particularly as more protracted outbreaks are identified.

**Summary**

**What is already known about this topic?**

Restaurant outbreaks of *Salmonella* with *Salmonella*-positive environmental samples might result in a higher proportion of *Salmonella-*positive food workers and longer outbreaks.

**What is added by this report?**

A protracted restaurant-associated outbreak of *Salmonella* Mbandaka in Michigan was identified through recursive case interviewing, asymptomatic employee testing, and environmental sampling. Multiple efforts to eradicate the organism failed, and the restaurant was permanently closed in 2018.

**What are the implications for public health practice?**

Coupling asymptomatic food worker testing and environmental sampling for *Salmonella* with whole genome sequencing of case isolates in suspected, protracted restaurant outbreaks of *Salmonella* enhances source identification. Exclusion or restriction of asymptomatic food workers with nontyphoidal *Salmonella* should be considered part of restaurant outbreak mitigation.
